# The Effect of Temperature on Trophic Discrimination of Stable Isotopes (^13^C and ^15^N) and Biokinetics in Common Carp (*Cyprinus carpio*, L. 1758)

**DOI:** 10.1002/jez.70086

**Published:** 2026-04-08

**Authors:** I. Kuklina, J. Kubec, P. Balzani, T. B. Meador, M. J. Kainz, M. Buřič, L. Veselý

**Affiliations:** ^1^ Faculty of Fisheries and Protection of Waters, South Bohemian Research Center of Aquaculture and Biodiversity of Hydrocenoses University of South Bohemia in České Budějovice Vodňany Czech Republic; ^2^ Biology Centre of the Czech Academy of Sciences Institute of Soil Biology and Biogeochemistry České Budějovice Czech Republic; ^3^ Faculty of Science University of South Bohemia in České Budějovice České Budějovice Czech Republic; ^4^ WasserCluster Lunz—Biologische Station Lunz am See Austria; ^5^ Danube University Krems—University for Continuous Education Krems an der Donau Austria

**Keywords:** biokinetic, carbon, carp, nitrogen, trophic discrimination factor

## Abstract

Stable isotope analysis (SIA) provides essential information toward a better understanding of trophic ecology. However, the interpretation of SIA results relies on assumptions about the trophic discrimination factor (TDF), which aims to improve the accuracy but may lead to bias. In aquatic ecosystems, most biota are poikilothermic organisms, thus temperature is one of the most important parameters affecting all biological processes, including the trophic discrimination of stable isotopes. Therefore, we conducted an experiment to establish TDF for freshwater fish under different temperature regimes (15°C, 25°C, and natural ambient pond temperatures from July to September). We used common carp (*Cyprinus carpio*, L., 1758) as a model organism. In the first phase of the experiment (weeks 1–6), all fish were fed a defined fish feed to establish a baseline isotopic signal for all individuals. In the second phase (weeks 7–18), fish were randomly divided into three temperature groups and were all fed different types of fish feed. Results indicated temperature‐dependent TDF, with nitrogen showing higher temperature dependency, where highest TDF were observed in the 15°C treatment and lowest in the 25°C treatment. In contrast, for carbon, an equilibrium of muscle isotopic values was reached only in the natural ambient temperature regime. Presumably, discrimination of nitrogen isotopes was related to metabolic turnover rates, where all values were significant, while for carbon, only significant values were found under natural ambient temperature conditions. These findings highlight the importance of considering environmental thermal conditions in evaluating stable isotope signatures in trophic studies.

## Summary

• This study demonstrates the effect of temperature on biokinetic parameters and trophic discrimination factors and highlights the importance of considering environmental thermal conditions in evaluating stable isotope signatures in trophic studies.

## 1 Introduction

Stable isotope analysis (SIA) is an important tool that has many applications across broad fields of study, including archeology, geology, physiology, ecology, and many others. In ecological studies, SIA has been widely used to trace material flow and quantify trophic interactions across ecosystems (Peterson and Fry 1987; Fry 2006; Boecklen et al. 2011). In ecology, SIA has enabled food web reconstruction, thereby providing a better understanding of trophic ecology (Fry 2006; Boecklen et al. 2011). It is well known that the stable isotope composition of consumers differs from their prey, as a consequence of the consumers’ metabolic processes that lead to relatively more of the lighter isotope being lost from the body as waste products (Fry 2006). This consumer–diet offset forms the mechanistic basis for trophic inference using bulk stable isotopes (DeNiro and Epstein 1978, 1981; Minagawa and Wada 1984). The stable isotope ratios of carbon (¹³/¹²C) and nitrogen (¹⁵/¹⁴N), which are commonly investigated for food web reconstruction, vary between consumer and their prey organisms, such that the former exhibits δ¹³C and δ¹⁵N values that are higher by approximately 1‰ and 2‰–4‰, respectively (Post 2002), meaning that consumers typically become isotopically enriched in the heavier isotope relative to their diet. However, these values represent generalized averages, and substantial variation in discrimination has been documented among taxa, tissues, and environmental contexts (McCutchan et al. 2003; Vanderklift and Ponsard 2003).

Based on such trophic enrichment, it is possible to assess dietary carbon sources (δ¹³C) and the trophic positioning (δ¹⁵N) of aquatic consumers relative to primary consumers. However, establishing these trophic relationships hinges on an a priori assumption of isotopic fractionation factors between consumers and their diet, a.k.a. the trophic discrimination factors (TDFs). Thus, interpretations of variability in stable isotope composition rely on the choice of TDF and, if incorrect for the investigated consumer, may lead to inaccurate conclusions (Nielsen et al. 2018). This is particularly critical because TDFs are routinely applied in mixing models and trophic position estimates to align consumer and source isotope values (Phillips et al. 2014). For example, the outcomes of Bayesian mixing models, which are robust models for SIA data interpretation, are highly sensitive to TDF values (Martínez del Rio et al. 2009; Moore and Semmens 2008; Nielsen et al. 2018; Post 2002). Even modest uncertainty in TDFs can propagate into substantially different dietary reconstructions (Bond and Diamond 2011; Phillips et al. 2014). Hence, assumptions of accurate TDFs in food web studies are critical for advancing our understanding of trophic ecology and broader impacts, such as the development of best management practices.

TDFs vary depending on biotic and abiotic conditions, including tissue type, taxon, feeding guild (e.g., carnivores vs. herbivores), food quality, habitat, salinity, temperature, and combinations thereof (Fry 2007; Veselý et al. 2024). Mechanistically, variation in δ¹⁵N has been linked to consumer growth, protein turnover, and nitrogen excretion pathways, whereas δ¹³C is strongly influenced by lipid dynamics and carbon routing during assimilation (Vanderklift and Ponsard 2003; Phillips et al. 2014). Large‐scale syntheses of isotope incorporation across taxa further demonstrate that isotopic turnover rates and tissue‐specific half‐lives scale predictably with body mass and differ markedly among tissues and major taxonomic groups (Thomas and Crowther 2015; Vander Zanden et al. 2015). In particular, isotopic turnover is generally slower in larger‐bodied organisms and in structural tissues such as muscle compared to metabolically active tissues, reflecting differences in growth rate, metabolic intensity, and protein replacement dynamics. These patterns reinforce that isotope incorporation is governed by physiological constraints and metabolic scaling rather than by a fixed temporal constant, and that discrimination and turnover dynamics must be interpreted within the context of organismal energetics and tissue‐specific processes (Thomas and Crowther 2015; Vander Zanden et al. 2015).

In aquatic ecosystems, most consumers and their prey are poikilotherms, and temperature is a key parameter affecting all biological processes (Brown et al. 2004; Dukes and Mooney 1999; Veselý et al. 2019). Because temperature regulates metabolic rate, growth efficiency, and isotopic turnover rate, it can indirectly alter discrimination by shifting the balance between anabolic and catabolic processes (Tieszen et al. 1983; Brown et al. 2004; Maitland et al. 2021). Variations in temperature amplitudes and the thermal optima of organisms can, directly and indirectly, affect individuals as well as whole communities, and have been shown to influence TDFs in fish (Bloomfield et al. 2011; Maitland et al. 2021). However, the effect of temperature on TDF remains poorly understood. For example, Canseco et al. (2021) suggested that increasing temperature decreases TDF of stable carbon and nitrogen isotopes in different ways among species, food source composition, or thermal history of consumers. Similarly, research on juvenile Pacific cod (*Gadus macrocephalus*, Tilesius, 1810) has shown that interactions between temperature and dietary quality can result in marked variations in TDF (Overmyer et al. 2008; Syväranta and Rautio 2010; Rogers et al. 2023).

Recent syntheses emphasize that TDFs reflect physiological state and energy allocation pathways, rather than representing fixed isotopic offsets between consumers and their diets (Stephens et al. 2022). In addition, dietary composition further interacts with temperature to shape TDF, which emerge from the combined effects of macronutrient routing, growth state, and metabolic balance rather than from fixed consumer–diet differences (Stephens et al. 2022). Lipid‐rich diets can reduce δ¹³C through preferential routing of ¹³C‐depleted lipids into storage pools, whereas protein‐rich diets and elevated rates of amino acid catabolism tend to increase δ¹⁵N via enhanced nitrogen excretion processes (Gannes et al. 1997; Sweeting et al. 2007; Stephens et al. 2022). In addition, dietary composition further interacts with temperature to shape TDF, with lipid‐rich diets often reducing ¹³C discrimination and protein‐rich diets enhancing ¹⁵N enrichment (Gannes et al. 1997; Sweeting et al. 2007). These interactions underscore the complexity of using isotopic data to infer trophic relationships in ecosystems subject to seasonal or climate‐induced temperature variation (Perga and Gerdeaux 2003, 2005; Jardine et al. 2006).

Therefore, the very commonly used TDFs established by Zanden and Rasmussen (2001) are unlikely to be broadly applicable across aquatic consumers. Similarly, the diet‐dependent discrimination framework proposed by Caut et al. (2009) was later criticized for experimental design limitations (Perga and Grey 2010). In addition, investigations of aquatic invertebrates by Brauns et al. (2018) suggested that food source C:N:P ratios and consumer–resource stoichiometric imbalances are strong predictors of TDF. Comparable patterns have also been reported for fish, indicating strong interspecific and context‐dependent variability in discrimination factors (Britton and Busst 2018; Mohan et al. 2016). Although phylogenetic approaches such as the R package SIDER have been developed to estimate TDFs (Healy 2017, 2018), these tools are currently limited to mammals and birds. Given these constraints, there is an urgent need to constrain the sensitivity of TDFs to changing environmental temperatures among poikilothermic consumers, particularly in the context of ongoing climate change.

Recent studies estimated TDFs of aquatic organisms under laboratory conditions (Blanke et al. 2017; Brauns et al. 2018; Glon et al. 2016; Veselý et al. 2024). These studies indicate that TDF is driven primarily by physiological and biochemical processes rather than fixed trophic offsets (the assumption that TDFs are constant values applied universally across consumers and conditions). Variation in δ¹⁵N was mainly associated with dietary protein quantity and quality, reflecting differences in amino acid catabolism and nitrogen excretion efficiency, whereas δ¹³C variability was linked to biochemical routing of dietary carbon, particularly the relative assimilation of lipids versus proteins and carbohydrates. In addition, consumer–diet stoichiometric imbalances (C:N:P) and growth rate emerged as key regulators of isotopic discrimination, with higher growth efficiency generally resulting in lower δ¹⁵N values. Additionally, a meta‐analysis performed by Canseco et al. (2021) suggested that nitrogen TDF decreases with increasing temperature, whereas the effect on carbon discrimination remained less consistent. Thus, we still lack a consensus on the effects of temperature amplitudes or fluctuations on TDFs across species, and a mechanistic understanding of specific consumer–diet processes is needed. This study focused on the effect of three different temperature regimes (15°C, 25°C, and a natural ambient temperature regime) on carbon and nitrogen TDFs in the common carp (*Cyprinus carpio* L., 1758), a species exposed to a wide range of water temperatures. We hypothesized that carbon and nitrogen TDF values (i) decrease with increasing temperature and (ii) fall between low and high temperature treatments under natural ambient conditions.

## 2 Material and Methods

Our experiment was conducted between April and September 2023 at the Research Institute of Fish Culture and Hydrobiology in Vodňany, Faculty of Fisheries and Protection of Waters, University of South Bohemia, Czech Republic. For experimental purposes, we used common carp (*C. carpio*) older than 1 year. Changes in fish size over the course of time in part 2 of this experiment are presented in results section in Table S1. All handling of organisms was conducted in accordance with the principles of the Institutional Animal Care and Use Committee (IACUC) of the University of South Bohemia, Faculty of Fisheries and Protection of Waters, Research Institute of Fish Culture and Hydrobiology, Vodňany, and complied with the EU‐harmonized animal welfare legislation of the Czech Republic.

The first part of the experiment lasted 6 weeks and was conducted in six tanks connected to a small recirculation system. Each tank measured 2600 × 420 × 190 mm (length × width × height) and was filled with aged tap water to a depth of 170 mm (approximately 150 L). Tanks were covered with transparent plastic lids to allow light penetration while preventing fish escape. Wooden spacers (450 × 10 × 10 mm; length × width × height) were placed between the lids and the tanks to facilitate gas exchange. Continuous aeration was provided in all tanks to prevent oxygen depletion. Fish were maintained at 20°C and fed daily ad libitum with Type I commercial feed (Table 1) to homogenize muscle stable isotope values across the experimental stock. This step was necessary to establish a common isotopic baseline, from which subsequent deviations could be attributed to experimental treatments. In this first phase, fish were fed a commercial flake feed for ornamental fish (Prodac Pondmix; Table 1). Tanks were cleaned every second day, and one‐third of the water volume was replaced with aged tap water maintained at 20°C. The photoperiod was set to 12 h light:12 h dark.

**Table 1:** Fish feed composition.

Feed	Feed type	Crude protein (g kg⁻¹)	Crude lipid (g kg⁻¹)	Crude fiber (g kg⁻¹)	Ash (g kg⁻¹)	δ^13^C	δ^15^N
**Prodac Pondmix**	Type I	105–110	20–25	~20	~20	−24.16 ± 0.03	6.7 ± 0.08
**Skretting TL 2 Tilapia**	Type II	320–360	50–80	< 50	70–90	−22.64 ± 0.49	3.2 ± 0.2

**Figure 1:**
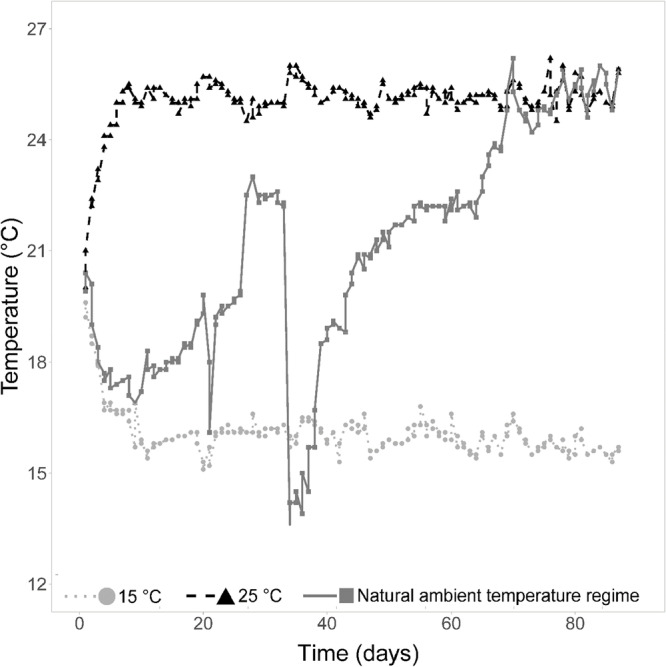
Temperature group treatments during part 2 of the experiment. Data are present as mean value temperature of given timepoint per temperature group.

After 6 weeks, fish were randomly assigned to three temperature treatments (15°C, 25°C, and a natural ambient temperature regime), which followed temperature fluctuations in a nearby pond. Fish were gradually acclimated to their target temperatures at a rate of 1°C per day. The second part of the experiment lasted 12 weeks, during which fish were fed exclusively with Type II commercial pellets (Skretting TL 2 Tilapia; Table 1). Each temperature treatment consisted of 60 individuals, which were randomly distributed among 6 aquaria (550 × 500 × 350 mm; length × width × height; filled by 80 L) connected to independent small recirculation systems. Aquaria were cleaned every second day, and one‐third of the system water volume was replaced with aged tap water adjusted to the corresponding treatment temperature. Water temperature was measured twice daily (morning and afternoon) in each temperature treatment (Figure 1).

At the beginning of the second experimental phase, 10 fish were euthanized, and their stable isotope values were used to define the baseline for subsequent analyses. To monitor temporal changes in muscle stable isotope composition, four fish per week were randomly sampled and euthanized throughout the experiment. Skinned dorsal muscle samples were dissected and stored at −30°C in a freezer (GN 3023‐22, Liebherr, Germany) until further processing. Fish were weighed to the nearest 0.1 g.

### 2.1 Stable Isotope Analysis

Prior to analysis, samples were freeze‐dried and ground to a fine, homogeneous powder. Then, approximately 0.6 mg of animal and fish food samples were weighed using a microbalance (Sartorius CPA2P, precision: 0.001 mg) into tin capsules.

Stable isotope analysis was performed at the Institute of Soil Biology and Biogeochemistry, Biological Centre Academy of Science (České Budějovice, Czech Republic). The analysis was performed using an EA‐Isolink CNSOH connected to a MAT253 Plus isotope ratio mass spectrometer via a Conflo IV device (ThermoFisher Scientific, Bremen, Germany). To control instrument stability, the muscle of the Northern pike (*Esox lucius*) of known isotopic composition was routinely analyzed during the analysis sequence, every nine samples. The *δ* values were normalized to the international VPDB and atmospheric N_2_ scales according to the measured values of the IAEA‐600 (caffeine) reference standard, which was analyzed at the beginning and end of each sequence. Results are expressed using the conventional *δ* notation and the analytical precision was < 0.1‰ for δ^13^C and < 0.3‰ for δ^15^N.

### 2.2 Statistical Analysis

#### 2.2.1 Growth‐Based Model

Prior to the analysis, we employed the correction for carbon as the range of C:N ratio in common carp muscle were between 3.5 and 6.1 (Post et al. 2007):

$$\delta^{13}C\;\mathrm{normalized}=\delta^{13}C\;\mathrm{untreated}-3.32+0.99*C:N\;\mathrm{ratio}$$

Where δ^13^ normalized is normalized δ^13^ carbon, δ^13^ untreated is the original value.

Later, we followed the approach described by Glon et al. (2016) to estimated δ^13^C, δ^15^N, and the metabolic rate of common carp muscle tissue at equilibrium (*δ_f_*), using the growth‐based model of Hesslein et al. (1993) based on Nonlinear Least Squares method:

(1) $$\delta_{t}=\delta_{f}+(\delta_{i}-\delta_{f})e^{-\left(k+m\right)t}$$

where *δ_t_* is the δ^13^C or δ^15^N value of common carp muscle tissue at given temperature group at time *t*. Consequently, *δ_f_* is the estimated δ^13^C or δ^15^N of common carp muscle tissues when reaching an equilibrium with their new diet, while *δ_i_* is the initial δ^13^C or δ^15^N of the tissue, *m* the metabolic turnover rate, and *k* the growth rate, that in turn can be calculated as:

(2) $$k=\frac{\ln\left(\frac{W_{t}}{W_{0}}\right)}{t}$$

where *W_t_* is the weight of the individual common carp at the time of the sampling (*t*), and *W*_0_ the initial weight. We used this equation as it allowed us to separate the relative contributions of common carp growth and metabolic tissue replacement to our isotope values, i.e., to quantify the half‐life of stable isotopes in each tissue (Fry and Arnold 1982).

For each temperature group in which both metabolic turnover rate and isotopic value of tissue in equilibrium were statistically significant, we further calculated the TDF (∆^13^C and ∆^15^N), which reflects the difference between the common carp *δ* values at the time they reach equilibrium (*δ_f_*) and that of their diet (*δ_d_*) as:

(3) $$∆=\delta_{f}-\delta_{d}.$$

We also estimated stable isotope half‐life, following the growth‐based model of Hesslein et al. (1993) modified by Glon et al. (2016), by setting *δ_t_* equal to the mid‐point between the measured *δ_i_* and the model estimate *δ_f_* and then solving for *t*:

(4) $$t=\frac{\ln\left(\frac{\delta_{t}-\delta_{f}}{\delta_{i}-\delta_{f}}\right)}{-(k+m)}$$

where *δ_t_*, *δ_f_*, *k*, and *m* are the parameters mentioned above.

#### 2.2.2 Fish Growth

The differences in fish growth among temperature groups were tested by generalized linear model with gamma distribution, where fish weight was used as the dependent variable and temperature together with time were independent variables. In addition, aquarium was used as a random factor. Later partwise post hoc tests were applied to assess the differences among the groups. All analysis was carried out in R software (version 4.3.3, R‐core team, 2025).

## 3 Results

**Figure 2:**
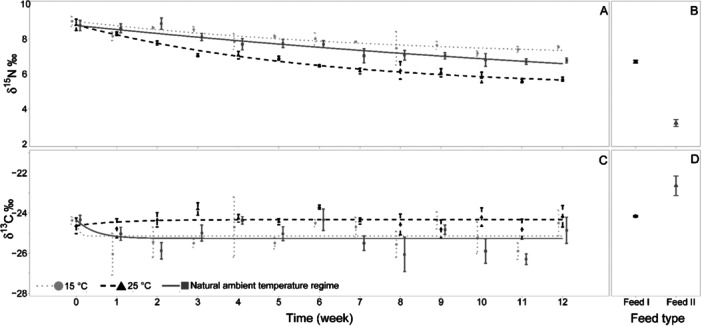
Mean ± SD of carbon (corrected δ^13^C) and nitrogen (δ^15^N) stable isotopes of carp muscle tissues over time during part 2 (A, C). The line represents predicted curves from non‐linear growth models for visual goodness of fit of the model. Panels (B) and (D) represent feed type isotopes value (mean ± SD). In part 2, feed type II was used, while feed type I was used in part 1 of the experiment.

The investigated temperature treatments exerted an effect on the TDFs exhibited by common carp, relative to their feed, and their metabolic parameters (Table 2, Figure 2). For carbon, the natural ambient treatment yielded results that were significantly different from zero, thus TDF and isotopic half‐life time were calculated only for this treatment. There was no significant difference from zero in the metabolic turnover rate model outputs for the two controlled temperature treatments (15°C and 25°C); therefore, other parameters which were estimated using values of metabolic turnover rate were not considered as significant due to bias given by non‐significant results of the metabolic turnover rate.

**Table 2:** Model‐estimated parameters of metabolic turnover rates, stable carbon and nitrogen delta values at equilibrium, and trophic discrimination factor (mean ± SD) for each temperature group (15°C, 25°C, and natural ambient regime).

Group	Metabolic turnover rate				Isotopic value of tissue in equilibrium				Isotopic half‐life time (days)	Trophic discrimination factor
	*Estimate*	*Low CI*	*High CI*	*p‐Value*	*Estimate*	*Low CI*	*High CI*	*p‐Value*		
δ^13^C										
15°C	−0.01	−0.01	0.03	0.18	−22.88	−25.66	−20.09	< 0.01	NA	NA
25°C	0.14	−0.01	0.02	0.51	−24.90	−26.07	−23.72	< 0.01	NA	NA
Natural ambient temperature regime	−0.01	−0.03	−0.01	< 0.01	−22.80	−23.73	−21.39	< 0.01	13.78	1.1 ± 0.24
δ^15^N										
15°C	0.02	0.0	0.05	< 0.01	7.08	6.04	7.65	< 0.01	20.21	5.32 ± 0.34
25°C	0.03	0.02	0.04	< 0.01	5.52	5.13	5.85	< 0.01	17.85	3.85 ± 0.18
Natural ambient temperature regime	0.03	0.01	0.05	< 0.01	7.18	6.42	7.95	< 0.01	24.38	4.49 ± 0.53

*Note:* Low CI and High CI denote the 95% confidential interval. Carbon isotope values were corrected due to high C:N ratio prior to statistical analysis.

All nitrogen model outputs were significantly different from zero, and muscle tissue reached isotopic equilibrium in all temperature groups. In addition, metabolic turnover rate was also significantly different from zero in all treatments. The highest TDF value was found in the 15°C temperature group, while the lowest TDF was found in 25°C. TDF of the natural temperature ambient group was in between the two controlled temperatures. Interestingly, isotopic half‐life did not follow this pattern, and the highest value was found in the natural temperature ambient group, while fish at 25°C exhibited the lowest value, and those at 15°C were in between.

**Figure 3:**
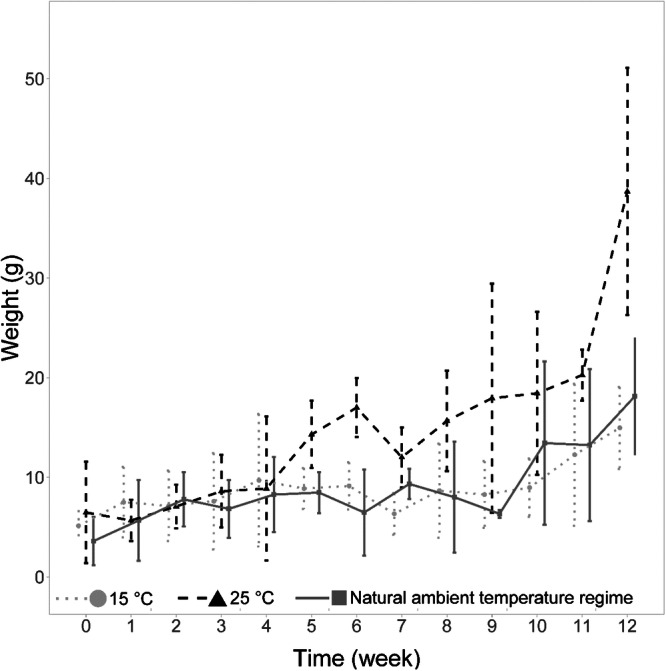
Common carp growth over time in part 2 of the experiment.

Fish growth was affected both by time and by temperature treatment. The interaction between time and temperature treatment was non‐significant (Table 3). Fish reared under 25°C reach the highest weight and were significantly different from the 15°C and natural ambient groups. On the other hand, there were no significant differences between 15°C and natural ambient groups in weight (Figure S1). The growth of common carp over the time among the temperature treatments are provided in Table S1 and Figure 3.

**Table 3:** Fish growth GLM model outputs.

	*F*‐value	*p*‐Value
Temperature group × time	0.86	0.42
Temperature group	18.64	< 0.01
Time	84.11	< 0.01

*Note:* Temperature groups represent common carp rearing in 15°C, 25°C and natural ambient temperature regimes. Time refers to day of sampling

## 4 Discussion

Ecological applications of stable isotope analysis rely on a mechanistic understanding of consumer–diet processes rather than assuming universal, constant isotopic enrichment between consumers and their diets. Our results demonstrate that TDFs and associated biokinetic parameters in common carp are sensitive to temperature, reinforcing the view that TDFs are dynamic properties emerging from physiological and biochemical processes. These findings are consistent with Canseco et al. (2021) and Britton and Busst (2018), who also reported a thermal dependency of TDFs in teleost fish and suggested that broad application of generalized TDFs is inaccurate and may lead to bias in data interpretation. This issue is particularly critical for Bayesian mixing models, which are highly sensitive to the choice of consumer TDF values (Bond and Diamond 2011; Martínez del Rio et al. 2009). Although recent advances have improved our ability to constrain TDF responses to environmental and ecological variables (e.g., Moore and Semmens 2008; Parnell et al. 2010; Stock and Semmens 2016; Canseco et al. 2021), uncertainty in TDF estimates remains one of the most influential sources of error in the interpretation of stable isotope patterns across food webs (Nielsen et al. 2018; Wolf et al. 2009).

Although the duration of the experiment was sufficient for isotopic incorporation to approach equilibrium (Vander Zanden et al. 2015; Thomas and Crowther 2015 and Veselý et al. 2024), carbon isotope equilibrium was not reached in the constant temperature treatments (15°C and 25°C). One possible explanation is that stable thermal conditions may have limited metabolic or biochemical processes influencing carbon incorporation, as discussed above. However, another limitation of the study is the relatively small difference in δ¹³C values between the two experimental diets (approximately 1.5‰), which may reduce the signal‐to‐noise ratio when detecting changes in consumer isotope values (Phillips et al. 2014). Additionally, the two feeds differed in nutritional composition, meaning that potential interactions between diet composition and temperature cannot be fully excluded in our experimental design. Nevertheless, despite these limitations, the study provides robust evidence that thermal variability influences biokinetic parameters and trophic discrimination dynamics in common carp.

In this study, both biokinetic parameters and TDFs exhibited systematic responses to thermal conditions. However, similar to the findings of Canseco et al. (2021), clear temperature‐dependent patterns in carbon TDFs were not consistently discernible under constant thermal regimes. Carbon isotope models did not converge for two temperature treatments with constant conditions (15°C and 25°C), and significant carbon TDF estimates were obtained only under the natural ambient temperature regime, which was characterized by temporal temperature fluctuations. In contrast, Britton and Busst (2018) reported a temperature‐related pattern in carbon TDFs for cyprinid fishes but did not observe a corresponding response in nitrogen TDFs, which contrasts with our results. In carbon, Britton and Busst (2018) observed increasing trend in common carp TDF reared in 18°C and 23°C (2.36 ± 0.15 and 2.70 ± 0.16, respectively). While the same authors reported slightly lower δ¹⁵N values in 18°C in comparison to 23°C in common carp. On the other hand, in two other cyprinid fish (*Squalius cephalus* L. 1758, *Carassius auratus* L. 1758), they found no temperature dependency in TDFs, suggesting species‐specific effect of temperature on TDFs. In the present study, nitrogen TDFs and associated biokinetic parameters showed a clear and consistent relationship with temperature across all treatments, in agreement with earlier experimental work demonstrating temperature effects on nitrogen isotope dynamics in ectothermic organisms (e.g., Barnes et al. 2007). The comparison with previously published results further supports our findings.

At present, there is no clear consensus in the literature regarding the dependence of carbon TDFs on temperature (Barnes et al. 2007; Britton and Busst 2018; Canseco et al. 2021). However, the significant carbon TDF response observed under the natural ambient temperature regime in our experiment suggests that thermal variability itself, rather than absolute temperature, may be an important driver of carbon isotope discrimination. This interpretation is consistent with theoretical and empirical work indicating that isotopic incorporation and routing are governed primarily by metabolic processes rather than fixed environmental thresholds (Martínez del Rio and Anderson‐Sprecher 2008; Thomas and Crowther 2015). Fish inhabiting temperate freshwater ecosystems experience pronounced seasonal temperature fluctuations, which are closely linked to shifts in energy allocation, lipid synthesis, and lipid storage (Henderson et al. 1988; Sheridan 1988). Such thermal dynamics may be necessary to maintain physiologically relevant patterns of carbon routing, as lipid deposition and mobilization strongly influence δ¹³C values in fish tissues (McConnaughey and McRoy 1979; Kiljunen et al. 2006; Post et al. 2007). Although the fish used in this study were 1+ individuals and therefore not reproductively active, exposure to fluctuating temperatures may still promote metabolic states conducive to carbon allocation into lipid pools, thereby affecting isotopic turnover and discrimination (Jobling 1994; Sweeting et al. 2007). In contrast, constant cold or warm experimental conditions may constrain fish to an artificial physiological state that limits the production and turnover of carbon‐derived compounds (e.g., fatty acids) and promotes isotopic equilibrium between muscle tissue and diet, as suggested by previous laboratory‐based isotope studies conducted under stable thermal regimes (Batten 2003; Jardine et al. 2006).

Finally, the negative estimates of the metabolic turnover parameter for carbon should not be interpreted as biologically negative metabolic rates. In isotopic incorporation models, this parameter represents a modification of growth‐dependent turnover rather than metabolism per se, and reflects the balance between tissue accretion and metabolic replacement (Hesslein et al. 1993; Martínez del Rio and Anderson‐Sprecher 2008). Negative values therefore indicate that isotopic incorporation proceeded more slowly than predicted by growth alone, suggesting reduced metabolic replacement of carbon relative to tissue accretion (Fry and Arnold 1982; Buchheister and Latour 2010). Such patterns are consistent with biochemical routing of dietary carbon into lipid or storage pools, which can decouple carbon isotope turnover from somatic growth and have been documented in previous isotope incorporation studies (McConnaughey and McRoy 1979; Gannes et al. 1997; Kiljunen et al. 2006; Sweeting et al. 2007). Importantly, the combined turnover term remained positive, ensuring biologically realistic isotopic dynamics, as required by isotope incorporation theory (Hesslein et al. 1993; Martínez del Rio et al. 2009).

## 5 Conclusion

Overall, our results corroborate and extend previous experimental studies on cyprinid fishes by demonstrating that temperature and thermal variability influence trophic discrimination in an isotope‐specific manner. These findings underscore the necessity of using empirically derived, context‐dependent TDFs when interpreting stable isotope data, particularly in freshwater ecosystems subject to pronounced seasonal temperature variation.

## Author Contributions

L.V. designed study and did statistical analysis. I.K. together with L.V. the first draft of the manuscript. I.K., M.B., and J.K. performed experimental work. I.K., M.B., J.K., and L.V. collected samples. TBM analyzed samples. L.V., M.B., J.K., M.J.K., T.B.M., and P.B. provided comments to the manuscript.

## Funding

Ministry of Agriculture of the Czech Republic via the National Agency for Agricultural Research (Project No. ZEMĚ QK21010131). MEYS CZ grant LM2015075 and SoWa Research Infrastructure (No. CZ.02.1.01/0.0/0.0/16_013/0001782).

## Conflicts of Interest

The authors declare no conflicts of interest.

## Supporting information


**Table S1:** Common carp growth over time in part 2 of the experiment. Data are presented as weight in grams (mean ± SD). **Figure S1:** Fish growth over the time regardless of temperature group (A) and within temperature group (B). In panel B, asterisks denote p‐values < 0.01 and NS as non‐significant (> 0.05).

## Data Availability

The datasets used and/or analyzed during the current study is available on https://zenodo.org/records/18183563.
