# Supplementary material for: The Effect of Temperature on Trophic Discrimination of Stable Isotopes (13C and 15N) and Biokinetics in Common Carp (Cyprinus carpio, L. 1758)
Source: J Exp Zool A Ecol Integr Physiol. 2026 Apr 8;345(6):535–44. doi: 10.1002/jez.70086 (PMC13255015; doi:10.1002/jez.70086)
Supplement: Supplementary file 1 — Table S1: Common carp growth over time in part 2 of the experiment. Data are presented as weight in grams (mean ± SD). Figure S1: Fish growth over the time regardless of temperature group (A) and within temperature group (B). In panel B, asterisks denote p‐values < 0.01 and NS as non‐significant (> 0.05). [file JEZ-345-535-s001.docx]

**The effect of temperature on trophic discrimination of stable isotopes (^13^C and ^15^N) and biokinetics in common carp (*Cyprinus carpio*, L. 1758).**

Kuklina, I.^1^, Kubec, J.^1^, Balzani, P.^1^, Meador, T.B.^2,5^, Kainz M.J.^3,4^, Buřič, M.^1^, Veselý, L.^1^

*^1^ University of South Bohemia in České Budějovice, Faculty of Fisheries and Protection of Waters, South Bohemian Research Center of Aquaculture and Biodiversity of Hydrocenoses, Zátiší 728/II, 38925 Vodňany, Czech Republic.*

*^2^Biology Centre of the Czech Academy of Sciences, Institute of Soil Biology and Biogeochemistry, České Budějovice, Czech Republic*

*^3^WasserCluster Lunz – Biologische Station, Dr. Carl Kupelwieser Promenade 5, 3293 Lunz am See, Austria*

*4Danube University Krems - University for Continuous Education, Dr. Karl Dorrek Straße 30, 3500 Krems an der Donau, Austria*

*^5^ University of South Bohemia in České Budějovice, Faculty of Science, České Budějovice, Czech Republic.*

Corresponding author: Lukáš Veselý – [veselyl@frov.jcu.cz](mailto:veselyl@frov.jcu.cz) , +420728486172

**Table S1.** Common carp growth over time in part 2 of the experiment. Data are presented as weight in grams (mean ± SD).

| **Group/day** | **0** | **7** | **14** | **21** | **28** | **35** | **42** | **49** | **56** | **63** | **70** | **77** | **84** |
| --- | --- | --- | --- | --- | --- | --- | --- | --- | --- | --- | --- | --- | --- |
| **15 °C** | 5.1 ± 3.18 | 7.5 ± 3.5 | 7.1 ± 3.5 | 7.6 ± 4.8 | 9.7 ± 6.6 | 8.9 ± 2.1 | 9.1 ± 2.4 | 6.3 ± 2.1 | 8.7 ± 4.7 | 8.3 ± 3.4 | 9.0 ± 2.9 | 12.3 ± 7.1 | 15.0 ± 4.1 |
| **25 °C** | 5.1 ± 3.18 | 5.7 ± 2.1 | 7.1 ± 2.2 | 8.6 ± 3.6 | 8.9 ± 7.2 | 14.3 ± 3.4 | 17.0 ± 3.0 | 12.0 ±3.0 | 15.7 ± 5.0 | 17.9 ± 11.5 | 18.4 ± 8.2 | 20.3 ± 2.6 | 38.7 ± 12.4 |
| **Natural ambient temperature regime** | 3.6 ± 2.4 | 5.7 ± 4.0 | 7.8 ± 2.7 | 6.8 ± 2.9 | 8.3 ± 3.8 | 8.5 ± 2.1 | 6.5 ± 4.3 | 9.3 ± 1.5 | 8.0 ± 5.6 | 6.3 ± 0.4 | 13.4 ± 8.2 | 13.2 ± 7.6 | 18.1 ± 5.9 |


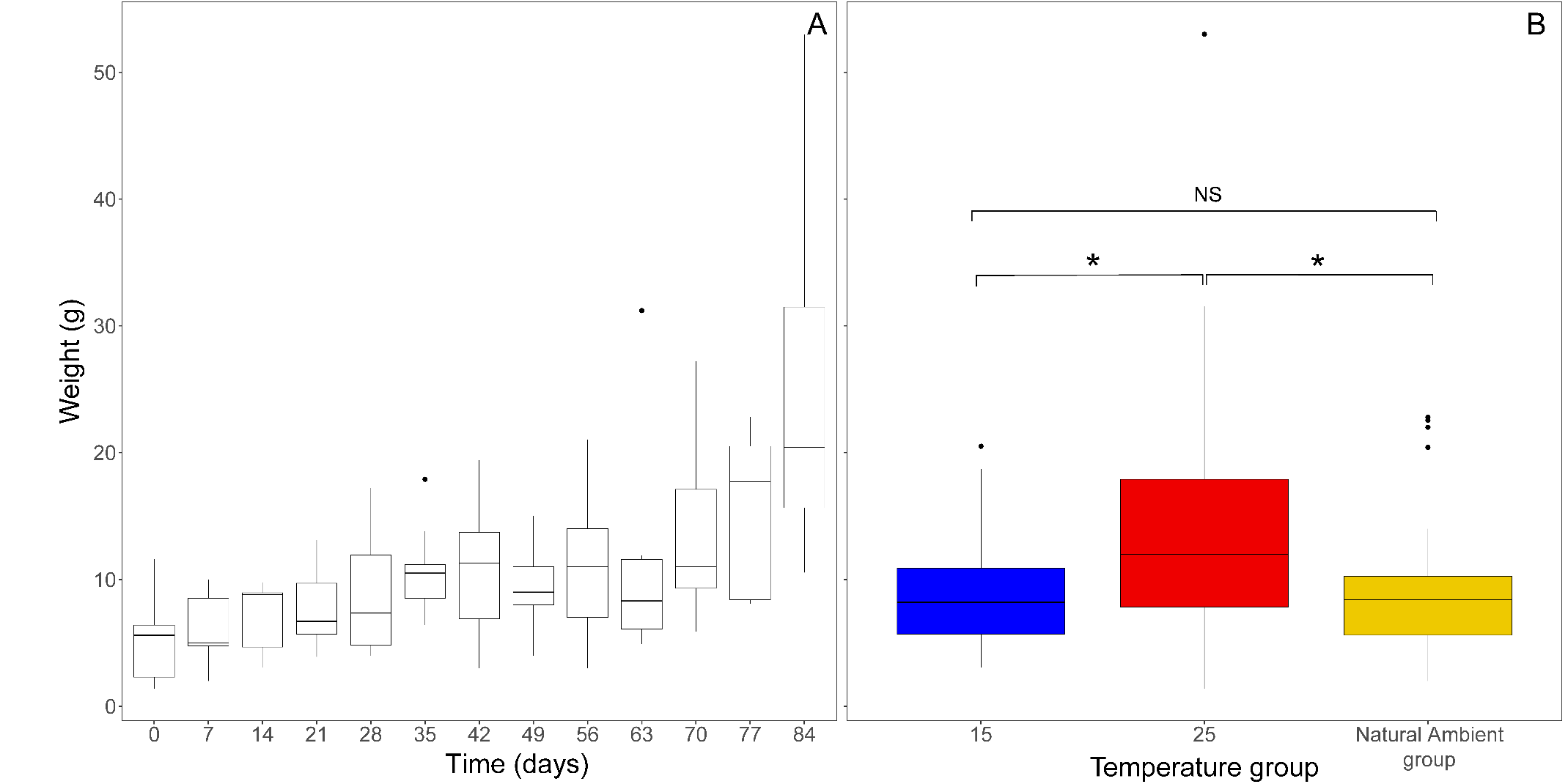


**Figure S1.** Fish growth over the time regardless of temperature group (A) and within temperature group (B). In panel B, asterisks denote p-values <0.01 and NS as non-significant (>0.05).
